# Early Detection of Keratoconus: Diagnostic Advances and Their Impact on Visual Outcomes: A Systematic Review

**DOI:** 10.3390/medicina62010042

**Published:** 2025-12-25

**Authors:** Evangelos Magklaras, Konstantinia Karamitsou, Vasilios F. Diakonis, Theodoros Mprotsis, Konstantinos T. Tsaousis

**Affiliations:** 1 Ophthalmology Department, General Hospital of Volos, 38222 Volos, Greece; emagklaras@hotmail.com (E.M.); konkaramitsou@gmail.com (K.K.); 2 2nd Ophthalmology Department, Metropolitan Hospital, 18547 Athens, Greece; diakonis@gmail.com; 3 Department of Biomathematics, School of Medicine, University of Thessaly, 41222 Larissa, Greece; tmprotsis@uth.gr

**Keywords:** keratoconus, early keratoconus, early diagnosis, corneal tomography, artificial intelligence, machine learning, visual outcome

## Abstract

*Background and Objectives*: Keratoconus is a progressive corneal ectatic disorder and a leading cause of corneal transplantation in developed countries. Early detection is critical for initiating timely interventions such as corneal cross-linking, which can halt disease progression and preserve long-term visual function. This review aims to synthesize current diagnostic approaches for early keratoconus detection and assess their clinical impact on visual outcomes. *Materials and Methods*: A comprehensive literature search was conducted across PubMed/MEDLINE, Web of Science, Google Scholar, Scopus and the Cochrane Library through September 2025. Search terms included “early keratoconus,” “subclinical keratoconus,” “forme fruste keratoconus,” “keratoconus detection,” “corneal topography,” “corneal tomography,” “anterior segment optical coherence tomography (AS-OCT),” “corneal biomechanics,” “artificial intelligence,” “genetic risk, “environmental factors”, and “machine learning.” Two independent reviewers analyzed the data. Studies were included if they investigated diagnostic modalities for early-stage keratoconus and discussed their relevance to visual outcomes. *Results*: One hundred and seven studies were included in the final review. Four diagnostic modalities demonstrated consistent clinical value: 1. corneal topography for assessing anterior surface irregularities; 2. corneal tomography, currently regarded as the gold standard due to its ability to detect early posterior elevation and pachymetric changes; 3. AS-OCT for epithelial and stromal profiling; and 4. biomechanical assessments, which evaluate corneal tissue stability prior to structural alterations. Artificial intelligence, when integrated with imaging data, enhances diagnostic sensitivity and standardizes interpretation across clinical settings. *Conclusions*: Early keratoconus detection is crucial for preserving vision; and integrating multimodal, AI-supported diagnostics into routine care—especially for high-risk groups—enhances accuracy, improves outcomes, and reduces progression rates of disease.

## 1. Introduction

Keratoconus (KC) is a bilateral, often asymmetric corneal disorder causing progressive thinning and conical protrusion, resulting in irregular astigmatism, myopia, and, in advanced cases, corneal scarring [1]. Keratoconus is a leading indication for corneal transplantation in Western countries and can be associated with other medical conditions. The initial detection of keratoconus can pose challenges, especially the forme fruste and the subclinical one [2]. The literature generally defines subclinical keratoconus as an eye that shows topographic abnormalities or patterns suspicious for keratoconus while maintaining a normal slit-lamp appearance, with the fellow eye already exhibiting clinical keratoconus. In contrast, forme fruste keratoconus is typically characterized by an eye that appears normal both on corneal topography and slit-lamp examination but is paired with a contralateral eye affected by manifest keratoconus. Early keratoconus is generally defined as the stage in which objective tomographic or biomechanical abnormalities consistent with ectasia are present, but clinical slit-lamp signs are minimal or absent and visual acuity remains largely preserved. This stage typically aligns with Amsler–Krumeich stage 1 or its equivalent in modern tomographic classification systems.

Patients often experience difficulty achieving adequate visual correction with spectacles and encounter variable reductions in visual acuity, image distortion, and increased sensitivity to glare and light [3]. The prevalence of keratoconus varies widely across different populations. The condition’s presentation and demographic patterns can offer insights into its broader epidemiological profile. Studies estimate the prevalence of keratoconus to range from 50 to 230 per 100,000 in the general population [4]. The prevalence of keratoconus is approximately 54.5 per 100,000 individuals in the United States [5]. Higher prevalence rates have been reported in certain ethnic groups, such as people of Middle Eastern, South Asian, and African descent [6]. Furthermore, first-degree relatives of KC patients also exhibited a significant prevalence of 7.7%, emphasizing the genetic and familial risk factors associated with the condition [7].

Keratoconus typically manifests during adolescence or early adulthood, progressing into the fourth decade of life [8]. Without early intervention, advanced keratoconus often requires corneal transplantation, which carries risks of graft rejection, infection, and high costs. Early detection enables less invasive treatments, such as CXL, which can stabilize the cornea and preserve vision, reducing both clinical and economic burdens [9]. This review provides a comprehensive evaluation of diagnostic modalities and genetic and environmental risk factors, while emphasizing the critical importance of early detection in optimizing visual outcomes.

In recent years, artificial intelligence (AI) has emerged as a promising adjunct in the detection and staging of keratoconus, particularly in its early or subclinical forms where conventional diagnostic tools may lack sensitivity. Advances in corneal imaging, machine-learning algorithms, and large annotated datasets have led to a rapidly expanding body of literature exploring AI-driven approaches for KC diagnosis. Although this review is not primarily focused on AI applications, the increasing number of studies dedicated to AI warrants a structured synthesis of their potential clinical utility, strengths, and limitations. Accordingly, we incorporated AI-related evidence to complement the evaluation of traditional diagnostic modalities and to highlight emerging technologies that may enhance early identification, risk stratification, and overall management of keratoconus.

## 2. Materials and Methods

### 2.1. Method of Systematic Review

This systematic literature review was conducted to synthesize current evidence on diagnostic techniques for the early detection of keratoconus and their impact on visual acuity outcomes. The electronic databases PubMed/MEDLINE, Web of Science, Google Scholar, Scopus and the Cochrane Library, were searched to identify relevant peer-reviewed articles published up to September 2025.

### 2.2. Study Selection

Two reviewers independently screened titles, abstracts, and full texts for eligibility. Inter-rater reliability was assessed using Cohen’s kappa (κ) using the IBM SPSS Statistics 29.0. The level of agreement between reviewers was substantial, with κ = 0.690 (standard error = 0.020). Agreement was statistically significant (T = 25.389, *p* < 0.001), based on 1354 screened records. Discrepancies were resolved through discussion and consensus, with arbitration by a third reviewer when necessary.

The search strategy incorporated various combinations of the following terms: “early keratoconus,” “subclinical keratoconus,” “forme fruste keratoconus,” “keratoconus detection,” “corneal topography,” “corneal tomography,” “anterior segment OCT,” “corneal biomechanics,” “artificial intelligence,” “genetic risk, “environmental factors”, “machine learning.”

Studies were included if they addressed diagnostic approaches applicable to the early stages of keratoconus, including forme fruste and subclinical forms, with an emphasis on technologies capable of detecting corneal changes before the development of overt clinical signs. Priority was given to peer-reviewed original studies and comprehensive reviews focusing on corneal imaging, biomechanical assessment, and AI-based diagnostic models.

Exclusion criteria included non-English articles, non-peer-reviewed publications, and studies that focused solely on advanced keratoconus.

The diagnostic modalities analyzed were grouped into four major categories: corneal topography, corneal tomography, anterior segment optical coherence tomography, and corneal biomechanical assessment. Each technique was evaluated in terms of its principles, strengths, limitations, and diagnostic accuracy as reported in the literature, with particular attention to their role in identifying early keratoconus and predicting progression.

### 2.3. Potential Sources of Bias

Several potential sources of bias may affect this review:

- Publication bias: Only published studies were included, which may exclude relevant data from unpublished or ongoing research.
- Language bias: Only English-language publications were evaluated, potentially omitting studies published in other languages.
- Selection bias: Study screening and eligibility assessment were performed by two independent reviewers; however, subjective judgment during this process may still introduce bias.
- Protocol bias: The review protocol was not registered in PROSPERO, an international database of prospectively registered systematic reviews, which may limit transparency and increase the risk of methodological deviations.

Despite these potential sources of bias, this systematic review was conducted in accordance with the Preferred Reporting Items for Systematic Reviews and Meta-Analyses (PRISMA) 2020 guidelines. This systematic review was not prospectively registered.

## 3. Results

**Figure 1:**
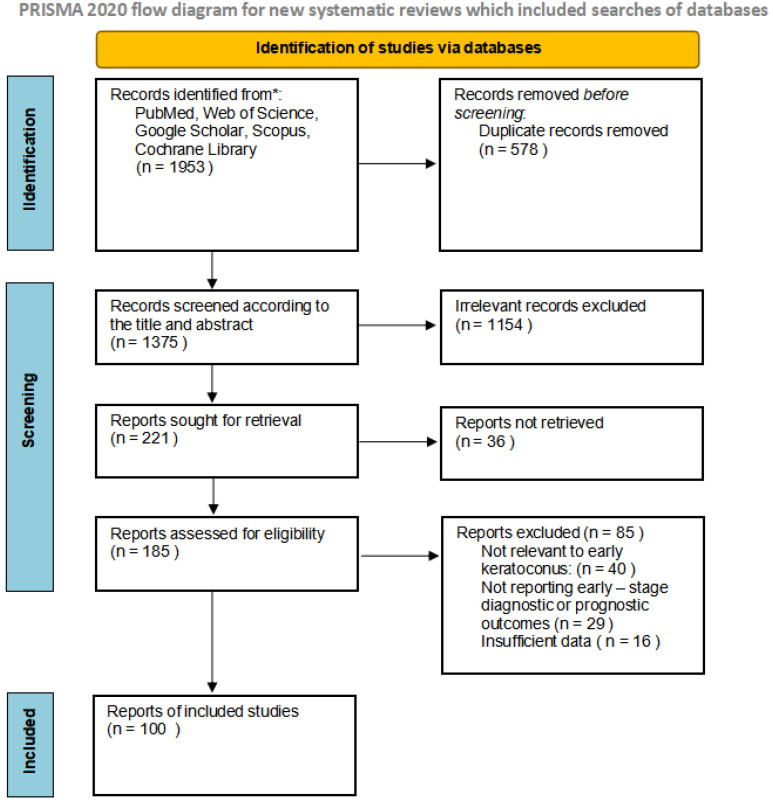
Flow diagram illustrating the study selection process.

A total of 1953 records were identified across the databases PubMed/MEDLINE, Web of Science, Google Scholar, Scopus and the Cochrane Library through September 2025. After removal of 578 duplicate records, 1375 records remained for screening. Titles and abstracts were evaluated, and 221 records were deemed potentially eligible and 36 were not retrieved. Following this stage, 85 records were excluded for not meeting the eligibility criteria. Ultimately, 100 studies met the predefined inclusion criteria and were incorporated into the qualitative synthesis (Figure 1). These studies were analyzed to summarize current evidence on diagnostic modalities for early keratoconus detection and their relevance to visual acuity outcomes.

### 3.1. The Importance of Early Diagnosis in Keratoconus

Early detection is paramount to halting keratoconus progression, maintaining visual acuity, and avoiding invasive interventions. Timely diagnosis allows for treatments like CXL, which strengthens corneal collagen, preventing further deterioration. Identifying keratoconus at its earliest, subclinical stage—before symptoms appear—is critically important, particularly in individuals being evaluated for refractive surgery. Early detection helps to avoid postoperative worsening of the disease and the onset of corneal ectasia, emphasizing the need for highly sensitive diagnostic methods capable of recognizing even subtle corneal abnormalities [10]. Early diagnosis also enables monitoring of high-risk groups, such as those with a family history or frequent eye rubbing, improving outcomes [7].

### 3.2. Impact on Visual Acuity

In populations with a high incidence of keratoconus (for example patients from countries with warm climates, with atopic diseases, adolescents and young adults) prompt and accurate diagnosis plays a pivotal role in effective disease management and prevention of progression [11]. Early detection directly correlates with better visual outcomes. Keratoconus progresses faster and is more aggressive in children and adolescents. Early detection and prompt treatment are essential to prevent severe vision loss and developmental impacts in children [12,13,14]. Patients who present late, especially with advanced disease are less likely to benefit from CXL and more likely to require invasive procedures like keratoplasty (corneal transplantation) which carry higher risks and longer recovery [15].

### 3.3. Diagnostic Methods for Early Detection of Keratoconus

Early keratoconus can now be detected more reliably through advanced imaging, corneal biomechanics, and AI-based tools, which often reveal changes that conventional topography cannot. Recent innovations also focus on making diagnostics more accessible and affordable.

#### 3.3.1. Corneal Topography

Corneal topography is classically defined as a non-invasive exploratory technique to analyze both qualitatively and quantitatively the morphology of the cornea. Corneal topography maps the anterior corneal surface, detecting irregular astigmatism and inferior steepening, hallmark signs of early keratoconus [16]. Corneal topography involves projecting light from a luminous object onto the cornea and analyzing the reflected image. There are several techniques used for this, including: the Placido disc method, slit-scanning technology, and the Scheimpflug imaging technique.

The Placido disc approach assesses only the anterior corneal surface, as it relies on the reflection of a projected series of concentric rings from the corneal epithelium. Unlike this technique, imaging systems based on slit-scanning or Scheimpflug principles generate three-dimensional representations of the cornea, allowing simultaneous evaluation of both anterior and posterior surfaces, as well as measurement of corneal thickness across its entirety. A centrally located camera captures the reflected image, which corresponds to the first Purkinje reflex. From this reflection, the curvature of the central 3 mm of the cornea is estimated, yielding Simulated Keratometry (Sim K) values. These rings are aligned with the visual axis rather than the center of the pupil. The spatial difference between the pupil center and the visual axis is referred to as angle kappa (K). Specialized algorithms analyze how the ring pattern is distorted to determine the corneal power at various points. While this method gives a precise assessment of the cornea’s refractive characteristics, it does not provide an accurate depiction of the cornea’s actual shape [17].

Slit scanning technology functions by analyzing the dimensions of a projected slit beam on the corneal surface. Initially, the Orbscan system utilized this method exclusively to evaluate corneal thickness, along with the anterior and posterior curvature of the cornea. In this system, elevation data were obtained directly, whereas curvature measurements were subsequently derived. The introduction of Orbscan II marked a significant advancement by integrating a Placido disc, allowing for direct acquisition of curvature data. The latest evolution, the Orbscan IIz, further enhances diagnostic capabilities by incorporating a Shack-Hartmann wavefront sensor within the Zyoptix workstation.

Scheimpflug imaging employs a rotating camera system to capture reflections from a bright slit beam as it traverses the corneal surface. This allows for the generation of a three-dimensional reconstruction of both anterior and posterior corneal elevation, from which pachymetric data are derived [18]. The Pentacam system utilizes both a single rotating camera and a static camera to acquire comprehensive corneal measurements. Current corneal topographers are based on one of these technologies: (i) systems based on the light reflection on the cornea, (ii) systems based on the projection of a slit light onto the cornea, and (iii) systems based on the asymmetric reflection of multicolor light-emitting diodes. Rabinowitz proposed diagnostic criteria for keratoconus, which were later refined to include a corneal power exceeding 47 diopters, the presence of irregular astigmatism characterized by an asymmetric bowtie pattern, and an inferior–superior (I–S) value greater than 1.4 diopters [19]. The I–S value is calculated by averaging five points along each hemi-meridian—located 3 mm from the corneal center and spaced at 30-degree intervals—and comparing the superior and inferior regions [20]. These indices were integrated into topography software systems to enhance early diagnosis. The TMS-1 corneal topographer incorporates software that evaluates eight quantitative indices per topographic map and generates a Keratoconus Classification Index (KCI), expressed as a percentage, to estimate disease severity.

#### Strengths

1. Sensitive Detection of Early Ectatic Changes.

Corneal topography, particularly Placido-based systems, is highly sensitive for detecting early or subclinical forms of keratoconus through quantitative indices such as the inferior–superior (I–S) value, skewed radial axis, and asymmetric bowtie patterns [21].

2. Non-Invasive and Rapid Acquisition.

The technique is non-contact, well-tolerated by patients, and enables rapid acquisition of high-resolution curvature maps, making it feasible for routine clinical screening.

3. Detailed Anterior Surface Analysis.

Topographic imaging provides precise curvature data of the anterior corneal surface, which is critical in evaluating regular and irregular astigmatism.

4. Quantitative and Reproducible Metrics.

Topography generates reproducible quantitative indices (e.g., simulated keratometry [SimK], keratoconus indices), facilitating objective assessment and longitudinal monitoring of corneal shape and disease progression [22].

5. Monitoring of Disease Progression and Treatment Response.

Serial topographic maps allow clinicians to monitor keratoconus progression and evaluate the efficacy of interventions such as corneal cross-linking.

#### Limitations

1. Limited Assessment of the Posterior Corneal Surface. Placido-disc systems do not provide direct measurements of posterior corneal elevation or true corneal thickness, which limits the ability to detect posterior ectatic changes that may precede anterior abnormalities.
2. Dependence on Tear Film Stability. Image quality and measurement accuracy are significantly affected by tear film irregularities, which may introduce artifacts or distort curvature maps [23].
3. Reduced Accuracy in the Peripheral Cornea. Placido-based topography provides the most reliable data within the central 3–5 mm of the cornea; peripheral measurements are less consistent and may lack clinical precision.
4. Susceptibility to Artifacts from Ocular Surface Pathologies. Corneal opacities, scarring, or surface irregularities can interfere with image acquisition by scattering light or distorting reflected mires, reducing diagnostic accuracy.
5. Inability to Provide Total Corneal Power. Since it does not account for posterior corneal curvature or corneal thickness distribution, topography alone does not yield total corneal refractive power, which may be clinically relevant in certain settings (e.g., IOL calculations, toric IOL alignment).
6. Requirement for Expert Interpretation. Interpretation of topographic maps, especially in borderline or subclinical cases, demands clinical expertise to avoid misclassification or overinterpretation in the absence of corroborative clinical signs.

#### 3.3.2. Corneal Tomography

Corneal tomography is a critical tool in the assessment and diagnosis of various corneal pathologies, particularly keratoconus and other forms of corneal ectasia. Corneal tomography is widely considered the gold standard for identifying early keratoconus. As a non-contact imaging modality, corneal tomography offers detailed and precise visualization of both anterior and posterior corneal surfaces. Based on the 2015 expert consensus by Gomes et al. it is broadly recognized as the reference standard for the early detection of keratoconus [24].

With advancements in imaging technology, particularly devices like the Pentacam and Scheimpflug systems, detailed mapping of the cornea has become more accessible and essential for clinicians. Due to its high sensitivity, it is particularly effective in identifying subtle corneal changes, establishing it as one of the most dependable modalities for early keratoconus detection [25,26,27]. The use of modern tomographic devices, such as the MS-39, has been shown to improve measurement precision in identifying keratoconus progression. The implementation of adaptive thresholds and repeated measures has led to better classification accuracy, which is necessary for tailoring appropriate management strategies [28].

Primarily, tomography utilizes high-speed acquisition technology that enhances both measurement accuracy and repeatability. Unlike reflection-dependent platforms, elevation-based imaging is less susceptible to artifacts caused by involuntary eye movements, resulting in higher-quality and more consistent data [29]. Moreover, it evaluates a broader area of the cornea compared to curvature-based topography [30], which facilitates improved assessment of peripheral corneal regions—areas often involved in advanced keratoconus and pellucid marginal degeneration. It is important to note that many contemporary devices now merge topographic and tomographic assessments, allowing clinicians to obtain both curvature and elevation data in a single scan, which enhances diagnostic precision.

A further methodological strength is that elevation-based techniques do not depend on the Gullstrand schematic eye model, a theoretical assumption inherent in videokeratography and other Placido-disc systems. This independence allows for more anatomically accurate surface measurements.

One of the most clinically valuable features of elevation-based tomography is its capacity to evaluate the posterior corneal surface. This is particularly relevant given growing evidence that posterior elevation changes are detectable in the early stages of keratoconus. Studies have consistently shown that even eyes classified as subclinical keratoconus (SCKC) exhibit statistically significant differences in posterior elevation compared to healthy corneas [31].

#### 3.3.3. Anterior Segment Optical Coherence Tomography (AS-OCT)

Optical coherence tomography (OCT) is an advanced imaging method that employs low-coherence interferometry with near-infrared light to generate high-resolution cross-sectional images, enabling precise assessment of corneal layer morphology and thickness distribution [32]. The shift from time-domain to spectral-domain (Fourier-domain) OCT systems has significantly enhanced imaging performance by offering faster acquisition speeds, improved axial resolution, and increased tissue penetration. These improvements are largely attributed to the use of shorter wavelength light sources. Current technology enables the acquisition of images with axial resolutions below 5 microns (classified as ultra-high resolution) and those slightly above 5 microns (high resolution). However, a limitation of spectral-domain OCT is its relatively shallow imaging depth compared to time-domain OCT, due to its shorter lateral scan range [33,34,35].

Swept-source OCT has further advanced the field by enabling the rapid capture of multiple longitudinal and transverse sections, allowing for the construction of three-dimensional reconstructions of the cornea, anterior segment, and iridocorneal angle [36]. Several high-quality AS-OCT systems are commercially available. OCT technology has been utilized successfully for anterior segment evaluation, offering several clinically relevant applications. Its capabilities allow for non-contact imaging, detailed visualization and analytics of anterior segment structures of the human eye on one device [37]. Its non-contact nature enhances patient comfort, making it suitable for frequent monitoring.

Swept-source optical coherence tomography (SS-OCT) is a high-speed imaging modality with excellent sensitivity, enabling the assessment of both anterior and posterior corneal surfaces, as well as detailed cross-sectional tomographic views of the cornea [38,39]. It offers high precision and reproducibility in measuring corneal thickness (pachymetry) across a broad area of the cornea. Moreover, SS-OCT has demonstrated the ability to reliably distinguish clinically diagnosed keratoconus from normal corneal profiles. The advanced SS-OCT system has been demonstrated to provide highly accurate and reproducible assessments of the anterior segment [40,41,42]. Studies have confirmed its reliability for various ocular biometric measurements, including corneal curvature, anterior chamber angle, and axial length [43,44].

SS-OCT produces corneal topography derived from elevation-based mapping techniques. The technique utilizes several quantitative parameters derived from the central 5 mm zone of the pachymetry map. These include: the minimum corneal thickness (Min), the difference between the minimum and maximum thickness values (Min–Max), the average thickness gradient between the superonasal and inferotemporal quadrants across concentric rings of 2 to 5 mm in diameter, and the standard deviation of epithelial thickness distribution (pattern standard deviation) [45]. In a study by Itoi et al., the ratio of anterior to posterior corneal surface area (As/Ps), as measured by anterior segment OCT, demonstrated high diagnostic performance for identifying forme fruste keratoconus (FFKC), with sensitivity and specificity values of 0.92 and 0.96, respectively, using a threshold of 0.99. These results were comparable to the Belin–Ambrósio deviation (BAD-D) index generated by rotating Scheimpflug tomography, which achieved sensitivity and specificity of 1.00 and 0.90, respectively, at a cut-off value of 1.33 [46]. Additionally, Scuderi et al. evaluated pachymetric indices derived from spectral-domain OCT (SD-OCT) for their effectiveness in detecting early keratoconus. The data presented in this study suggest that anterior segment OCT may serve as a valuable adjunctive tool for the early detection of keratoconus. Among the evaluated parameters, the C1–C2 index exhibited particularly strong diagnostic accuracy, C1 represents the average corneal thickness within a 1 mm radius around the thinnest point, while C2 refers to the average thickness measured at diametrically opposite locations relative to C1 [47].

Anterior segment optical coherence tomography offers improved diagnostic accuracy over elevation-based imaging techniques, particularly in cases involving corneal haze or scarring [48]. In scenarios where central corneal scarring is present, devices like the Orbscan II often underestimate corneal thickness, whereas AS-OCT continues to deliver precise and reliable pachymetric assessments. A notable advantage of AS-OCT in identifying early-stage keratoconus is its capacity to visualize the corneal epithelium in detail. This allows for comprehensive epithelial thickness mapping, which is critical for detecting early epithelial remodeling—a feature not typically captured by elevation-based corneal imaging methods [49,50]. OCT is a costly, typically non-portable diagnostic tool that necessitates trained personnel to obtain reliable corneal imaging. Additionally, its performance can be compromised by poor patient cooperation, and when used in isolation, it may not be sufficient for detecting early keratoconus. Therefore, it is often used alongside other imaging technologies or artificial intelligence-based approaches to improve diagnostic precision [47,51].

#### 3.3.4. Corneal Biomechanical Assessments

Corneal biomechanical characteristics describe the cornea’s behavior under applied forces. These measurable properties play a vital role in the early identification of keratoconus [52,53]. Assessing corneal biomechanics in vivo remains a complex task, with only two commercially available devices currently used to support the diagnosis of keratoconus.

The Ocular Response Analyzer (ORA) enables real-time evaluation of corneal biomechanical behavior [54]. The device uses a precisely calibrated air puff to temporarily deform the cornea, while infrared sensors detect the applanation events as the cornea moves inward and then outward. This process generates two pressure readings—one during the initial indentation and another as the cornea resumes its normal curvature. The difference between these two values is termed corneal hysteresis (CH), reflecting the cornea’s viscoelastic properties and its capacity to dissipate and absorb energy. CH is altered in various ocular and systemic diseases, indicating changes in corneal biomechanics [55]. Another parameter derived from the ORA is the corneal resistance factor (CRF), which estimates the overall biomechanical strength and resistance of the corneal tissue to deformation. In keratoconic eyes, both CH and CRF are typically reduced, correlating with increased disease severity [56]. These findings indicate a diminished ability of the cornea to resist mechanical stress and absorb energy as keratoconus progresses. However, despite their clinical value, neither CH nor CRF alone reliably distinguishes early keratoconus from normal corneas, limiting their diagnostic utility in borderline or mild cases [57].

The Corvis ST is a non-contact diagnostic tool that evaluates corneal biomechanics through high-speed dynamic Scheimpflug imaging. Recent research using the Corvis ST device demonstrated that novel parameters, particularly the stiffness parameter at first applanation (SP—A1) and the Corvis Biomechanical Index (CBI), can reliably distinguish subclinical keratoconus (SKC) from normal eyes, with SP—A1 achieving an AUC of 0.753 and CBI an AUC of 0.703 [58,59].

Brillouin microscopy is a cutting-edge, non-contact imaging method that evaluates corneal biomechanics by examining how light interacts with intrinsic acoustic waves—specifically, phonons—within the tissue. When near-infrared laser light is applied to the cornea, it undergoes frequency modulation as it scatters off these phonons. This subtle frequency alteration, typically in the gigahertz (GHz) range, can be translated into mechanical stiffness values expressed in gigapascals (GPa), allowing for quantitative assessment of tissue elasticity. The collected data is then visualized as a Brillouin elasticity map, offering spatial insight into the biomechanical characteristics of the cornea. Motion tracking (MT) Brillouin parameters independently distinguished subclinical keratoconus cases from healthy controls, without the need for integration with morphological data. This level of diagnostic performance contrasts with earlier Brillouin-based research, where initial-generation metrics showed limited ability to separate even more progressed keratoconus stages from normal eyes [60].

Brillouin imaging has demonstrated that corneal biomechanical asymmetry and inhomogeneity increase with keratoconus progression. In early disease, the mean Brillouin shift at the cone surpasses corneal thickness and curvature in diagnostic performance. Combined with morphological data, it may improve screening and preoperative risk assessment [61]. However, its current diagnostic accuracy across disease stages remains limited [62].

### 3.4. Artificial Intelligence (AI) in Keratoconus

Artificial intelligence (AI), particularly machine learning (ML) and deep learning (DL) methods, has emerged for improving diagnostic accuracy across a range of conditions, including keratoconus [63,64,65]. In early keratoconus detection, artificial intelligence techniques have shown improved diagnostic performance, exhibiting high sensitivity and specificity, especially when combined with imaging modalities like corneal topography, tomography, and biomechanical evaluations [66,67,68,69].

A recent systematic review and meta-analysis on keratoconus and artificial intelligence (AI) showed that AI algorithms can effectively distinguish KC eyes from healthy controls by analyzing data obtained from different corneal imaging platforms [70]. To address device dependency, future studies should explore cross-device harmonization techniques, such as data normalization across platforms, domain adaptation methods (e.g., adversarial training to align feature distributions from different devices), and transfer learning approaches (e.g., fine-tuning pre-trained models on new device-specific datasets) to enhance model generalizability However, since these AI models are tailored to specific imaging devices with varying input features and design objectives, they are not directly interchangeable. Artificial intelligence models such as feedforward and convolutional neural networks are widely utilized for the detection of keratoconus, with neural networks in particular showing consistently high diagnostic performance [71,72,73]. According to Lavric et al., the effectiveness of neural networks in keratoconus detection is largely attributed to their capacity to analyze topographic data from both eyes simultaneously, thereby improving their ability to distinguish between normal and diseased corneas [74]. Despite these promising results, most existing AI studies rely on retrospective data, often collected under non-uniform conditions and without external validation. This reliance on historical, retrospective datasets introduces biases, such as selection bias from non-representative patient cohorts, and limits real-time applicability in medical practice, where prospective, multicenter validation is essential for ensuring models perform reliably on diverse, unseen clinical data. This limits the generalizability of their findings and reduces their current applicability to routine clinical practice.

Furthermore, current research often lacks thorough assessments of deficiencies, including inadequate dataset selection criteria (e.g., imbalanced classes or small sample sizes leading to overfitting), limited model construction details (e.g., hyperparameter tuning methods), and insufficient validation approaches (e.g., k-fold cross-validation or hold-out testing on external cohorts). Addressing these gaps is crucial for advancing AI in keratoconus diagnostics.

#### 3.4.1. Key AI Architectures

Several AI architectures have been applied to keratoconus detection, each exploiting different aspects of corneal imaging data:

- Convolutional Neural Networks (CNNs) is the most common architecture. In recent years, convolutional neural networks (CNNs)—a class of deep learning models designed to interpret images and other two-dimensional data—have achieved remarkable success across numerous medical and non-medical domains. A typical CNN architecture consists of two main components [75,76]. The convolutional layers serve as the feature-extraction mechanism, automatically identifying relevant spatial patterns in the input images [72,75]. This automated extraction differs fundamentally from traditional machine-learning approaches, where feature selection depends heavily on human-defined parameters [77,78]. Following the convolutional block, a fully connected layer integrates the extracted features to perform the final classification task and determine the diagnostic category. There are a few published studies which have applied CNN-based models to the detection of keratoconus, although only three specifically focused on identifying subclinical disease. These studies drew on datasets obtained from several imaging platforms, including Pentacam, Orbscan, and OCT, demonstrating the versatility of CNNs across different corneal imaging technologies [79,80,81,82]. However, most CNN approaches do not report strategies for mitigating overfitting—such as data augmentation, cross-validation, or dropout—making it difficult to evaluate how stable the models would be on unseen clinical datasets. Compared to other architectures, CNNs excel in handling image-based inputs but are more susceptible to overfitting due to their high parameter count, particularly in small datasets; they also offer limited inherent explainability without additional tools like saliency maps.
- Artificial neural networks—particularly multilayer perceptrons (MLPs)—have also shown impressive diagnostic capabilities in keratoconus research. Several investigations have documented very high performance, with some reporting sensitivities approaching 100% [71]. Neural-network-based models are frequently employed, most commonly using corneal topography as the primary input. Among these, the study by Fisher and colleagues achieved the strongest results, reporting perfect sensitivity and specificity for distinguishing keratoconic eyes from healthy controls [83,84]. Nevertheless, these models rarely include explainability components or sensitivity analyses. Without such tools, it remains unclear which specific topographic or tomographic parameters drive the model’s decisions, which reduces transparency for clinical adoption. MLPs are computationally simpler than CNNs and less prone to overfitting in tabular data scenarios but perform worse on raw images and lack the spatial hierarchy learning of CNNs; their black-box nature similarly hinders explainability.
- Using the Harvard Dataverse data, sequential forward-selected random-forest models accurately differentiated keratoconus severity levels, yielding 98% accuracy for class 2 and 95% for class 4 [85]. In a comparative analysis of corneal topography and biomechanical data from Arkadiusz Syta, from all tested machine-learning approaches, random forests yielded the most reliable results, reaching an overall mean accuracy near 96%. Depending on the input features, accuracy values ranged from 93% to 98%. The highest performance was recorded with the Belin/Ambrósio Enhanced Ectasia Display features, where the model achieved 98% accuracy [86]. Although random forests are less prone to overfitting than neural networks, most current studies do not provide details regarding external testing, dataset balancing, or cross-device validation—factors necessary for assessing model robustness. Random Forests provide better explainability through feature importance rankings compared to neural networks and are robust to overfitting via ensemble methods, but they underperform CNNs on complex image data and require careful feature engineering.
- Support vector machines (SVM) have enabled the creation of tools such as the Fourier-based keratoconus index that can reliably flag subclinical keratoconus [65,87,88]. Models using cubic kernels combined with elevation data consistently show strong diagnostic ability, attaining roughly 96.6% accuracy when contrasting keratoconus with normal corneas [88,89]. Despite their strong performance, SVMs remain limited by dependence on carefully selected features, and current studies often lack information on feature-selection reproducibility, external validation, or model calibration across datasets. SVMs offer good generalization with lower overfitting risk in high-dimensional spaces than MLPs but are less scalable for large datasets compared to Random Forests and lack the automatic feature extraction of CNNs; explainability can be improved via kernel interpretations but is not standard.

Performance metrics across these architectures (e.g., sensitivity, specificity, accuracy) are reported from original sources as cited, but inconsistencies arise from varying study designs, such as different validation splits or class imbalances. For instance, CNNs often report AUC values above 0.95 in internal validation but drop in external tests, highlighting the need for standardized reporting. No table is included here, but if present elsewhere, it should align methodological details (e.g., training/validation splits, overfitting mitigation) with those of other diagnostic methods for consistency.

#### 3.4.2. Input Features and Training Data Sources

AI models in keratoconus research utilize a wide range of input data depending on the imaging device:

Topography: Axial/sagittal curvature maps, tangential maps, anterior elevation, posterior elevation.

Tomography (Scheimpflug): Pachymetric progression, Belin/Ambrosio indices, corneal thickness spatial profiles.

AS-OCT: Epithelial thickness distribution, stromal reflectivity patterns.

Biomechanics: CorVis ST metrics (DA ratio, integrated inverse radius), ORA metrics (corneal hysteresis, corneal resistance factor).

The heterogeneity of device-specific parameters explains why AI models are not directly interchangeable across imaging platforms. However, the absence of domain-adaptation strategies or cross-device harmonization methods means that current models remain device-locked, limiting their real-world deployment where patients may be imaged across different technologies. Regarding subclinical keratoconus detection, AI models leverage epithelial features (e.g., thinning patterns in AS-OCT), posterior features (e.g., elevation asymmetries in tomography), and biomechanical features (e.g., reduced hysteresis in ORA) through integrated multi-modal inputs. For example, CNNs can learn compensatory epithelial thickening over posterior ectasia, while Random Forests rank biomechanical stiffness as key predictors, though more studies are needed to dissect these contributions via ablation experiments.

#### 3.4.3. Clinical Applicability

AI has the potential to transform keratoconus screening and monitoring by providing:

- Automated, operator-independent risk assessment;
- Improved detection in borderline cases;
- Standardized interpretation across clinicians and centers;
- Valuable support in high-volume screening programs and teleophthalmology.

As the number of input cases increases, the AI system’s predictive accuracy improves through continuous learning, gradually adapting and refining its output based on accumulated experience. The application of machine learning models in the clinical management of keratoconus has the potential to significantly enhance the efficiency and accuracy of both diagnosis and disease monitoring. By facilitating earlier detection and more individualized follow-up, these technologies may ultimately contribute to the prevention of disease progression and the reduction in vision loss in affected patients [90]. Nonetheless, meaningful clinical adoption requires transparent AI systems. No existing keratoconus AI model incorporates Explainable AI (XAI), leaving clinicians without insight into the reasoning behind model predictions. XAI techniques, such as SHAP values for feature attribution or LIME for local explanations, are essential for medical applications to build trust and ensure regulatory compliance. Future research must address this gap, along with multicenter validation, standardized reporting of performance metrics, and evaluation across diverse populations to ensure reliability and clinical safety. Claims about AI precision and diagnostic standardization remain provisional without evidence from multi-site trials and prospective studies, which are needed to validate generalizability and mitigate biases.

### 3.5. Genetic Screening for Early Keratoconus Detection

Keratoconus is a degenerative eye disorder characterized by genetic variability and a multifactorial origin, most often occurring without a clear pattern in the general population. A notable percentage of patients—ranging from approximately 5% to as high as 23%—have relatives who are also affected, suggesting a hereditary component. The condition can be inherited through either an autosomal dominant or recessive mechanism. In dominant inheritance, various clinical forms may appear, often showing incomplete penetrance, meaning not all individuals who carry the gene will display symptoms [91]. Keratoconus genetic heterogeneity and complexity have made pinpointing causative genes difficult through traditional linkage and candidate gene approaches [6,92]. A systematic review and meta-analysis identified several single-nucleotide polymorphisms (SNPs) that may contribute to the risk of developing keratoconus, particularly in individuals of European ancestry. Among these, specific genes such as FOXO1, COL5A1, FNDC3B, IMMP2L, ZNF469, and COL4A4 have shown consistent associations. Some of these genetic variants were initially discovered through genome-wide association studies, while others emerged from candidate gene research. Although their precise functional roles remain to be fully understood, these findings suggest a strong genetic component in the pathogenesis of keratoconus [93]. Knowing that a family member is affected by keratoconus can raise clinical suspicion and prompt earlier screening, especially in challenging diagnostic cases. Although genetic testing remains costly and is not routinely performed in clinical settings, a positive family history can be a valuable clinical indicator for ophthalmologists. It enables earlier diagnosis of the disease, which in turn allows for earlier intervention and, consequently, improved visual outcomes for the patient.

### 3.6. Environmental Risk Factors and Their Role in Early Detection

Repetitive eye rubbing, especially when it is done with the knuckles, is recognized as a major independent risk factor contributing to the onset of keratoconus [94,95]. Recent meta-analytical data indicate that individuals who frequently rub their eyes have an increased risk of developing the condition compared to those who do not [96]. Additionally, habitual eye rubbing is observed in nearly 50% of patients diagnosed with keratoconus [97]. While several small-scale studies have reported associations between keratoconus and allergic conditions such as atopy or asthma, the most robust evidence stems from larger population-based studies [98]. One of the most comprehensive studies to evaluate individual-level risk factors for keratoconus was carried out in Iran by Naderan et al. (2017) [99], involving a cohort of 885 patients diagnosed with the condition. This sample size is comparable to that of a retrospective chart review conducted in Israel by Merdler et al. (2015) [98]. Both studies identified a consistent association between keratoconus and a history of allergic disease, including asthma.

Incorporating genetic screening into routine clinical practice may facilitate the early identification of individuals at increased risk for keratoconus, particularly when environmental risk factors—such as chronic eye rubbing and prolonged contact lens wear—are also present. Recognizing and addressing these modifiable factors at an early stage enables ophthalmologists to intervene sooner, potentially slowing or halting disease progression. This proactive, integrative approach not only improves the likelihood of early diagnosis but also optimizes visual outcomes and reduces the long-term burden associated with advanced keratoconus.

### 3.7. Socioeconomic Barriers to Early Diagnosis

There are some studies that provide valuable insight into the socioeconomic determinants of keratoconus severity at presentation. Their findings highlight that individuals from lower socioeconomic backgrounds tend to present with more advanced disease, likely due to delayed access to ophthalmologic care. This underscores the importance of implementing early detection strategies, particularly in underserved populations, to preserve visual acuity and improve long-term visual outcomes [99]. A large cohort study highlighted the significant influence of socioeconomic determinants—such as insurance status and geographic residence—on access to keratoconus management, particularly corneal collagen cross-linking (CXL). Individuals residing in socially disadvantaged areas were found to have reduced rates of receiving treatment, underscoring the critical impact of social and structural factors on healthcare equity in keratoconus care [100].

## 4. Discussion

The early diagnosis of keratoconus represents a pivotal clinical opportunity that can significantly influence visual outcomes. Evidence from this review indicates that patients identified at subclinical or forme fruste stages maintain superior visual acuity compared to those diagnosed after manifest disease develops. This temporal relationship is particularly pronounced in pediatric and adolescent populations, where keratoconus progresses more rapidly. Early intervention, especially with corneal cross-linking, can effectively arrest disease progression, whereas delayed diagnosis often necessitates corneal transplantation, with associated risks of graft rejection, infection, and prolonged rehabilitation.

No single diagnostic modality captures the full spectrum of early disease changes. Corneal topography remains a valuable first-line, non-invasive screening tool for anterior surface irregularities, while corneal tomography reveals subtle posterior elevation and pachymetric changes. Anterior-segment optical coherence tomography enables high-resolution mapping of epithelial architecture, detecting compensatory thickness alterations in early ectasia. Functional biomechanical measurements, derived from air-puff deformation and emerging light-scattering technologies, identify underlying tissue instability even when morphology appears normal. Combining these imaging and biomechanical data through advanced machine-learning algorithms enhances diagnostic accuracy and reduces subjective variability.

Technical sophistication alone, however, is insufficient. Socioeconomic and geographic barriers can delay diagnosis and treatment, increasing the likelihood of advanced disease at presentation. Equitable access to diagnostics, tele-ophthalmology networks, and targeted screening programs for high-risk populations is essential to maximize the benefits of early detection. Integrating cutting-edge technology with inclusive public health strategies can facilitate proactive, personalized care that preserves vision and improves quality of life for patients at risk of keratoconus.

This review has several limitations. Included studies exhibited substantial heterogeneity in study populations, imaging modalities, and definitions of early keratoconus, which may affect generalizability. Only published studies in English were considered, introducing potential publication and language bias. Study selection was performed by two independent reviewers, but subjective judgment cannot be entirely excluded. Additionally, a formal meta-analysis was not conducted due to variability in reported outcomes. Despite these limitations, the review synthesizes current evidence and highlights emerging diagnostic strategies for early keratoconus detection.

### 4.1. Diagnostic Technology Evolution and Visual Impact

The progression from basic corneal topography to advanced multimodal imaging has fundamentally transformed our capacity to detect keratoconus before visual compromise occurs. Traditional Placido-based topography, while accessible and widely available, primarily identifies anterior surface irregularities that represent relatively advanced disease stages. The limitation of this approach becomes evident when considering that posterior corneal changes, which are often the earliest detectable abnormalities, precede anterior surface manifestations by months or years.

Corneal tomography has emerged as the current gold standard precisely because it captures these early posterior elevation changes that correlate with better visual prognosis when treated promptly. This technological advancement has shifted the diagnostic paradigm from reactive identification of established disease to proactive detection of at-risk corneas. The integration of anterior segment optical coherence tomography adds another dimension to early detection by revealing epithelial thickness variations that represent compensatory mechanisms preceding topographic abnormalities. This capability to detect subclinical epithelial remodeling provides an additional temporal window for intervention, potentially preserving visual function that would otherwise be compromised by progressive stromal changes.

### 4.2. Functional Assessment Through Biomechanical Analysis

Corneal biomechanical assessment represents a paradigm shift toward functional evaluation of corneal integrity rather than purely morphological analysis. The reduction in corneal hysteresis and resistance factor observed in keratoconic eyes reflects fundamental changes in tissue mechanical properties that precede structural alterations visible on topographic imaging. This functional approach to early detection offers value in borderline cases where traditional imaging may appear normal despite underlying biomechanical compromise. The clinical significance of biomechanical parameters extends beyond diagnostic utility to prognostic value. Corneas with reduced biomechanical stability are more likely to experience rapid progression, making early identification through devices like the Corvis ST and Ocular Response Analyzer crucial for timely intervention. The correlation between biomechanical parameters and disease severity provides clinicians with objective metrics for assessing progression risk and optimizing treatment timing.

### 4.3. Artificial Intelligence and Enhanced Diagnostic Precision

The integration of artificial intelligence in keratoconus diagnosis represents a significant advancement in achieving consistent, objective, and highly accurate early detection. Machine learning algorithms demonstrate particular strength in analyzing complex, multidimensional datasets that incorporate topographic, tomographic, biomechanical, and epithelial thickness parameters simultaneously. This comprehensive approach achieves diagnostic high accuracy, significantly surpassing individual diagnostic modalities. The clinical value of AI-enhanced diagnosis extends beyond improved accuracy to standardization of interpretation. By reducing subjective variability in borderline cases, AI systems enable more consistent identification of early disease, ensuring that patients receive timely intervention regardless of individual clinician experience. This standardization is particularly valuable in screening programs and telemedicine applications where access to specialized expertise may be limited.

### 4.4. Addressing Healthcare Disparities in Early Detection

The review reveals significant socioeconomic barriers that impede early keratoconus detection, particularly in underserved populations. Patients from lower socioeconomic backgrounds consistently present with more advanced disease, reflecting delayed access to specialized ophthalmologic care. This disparity in presentation timing directly translates to worse visual outcomes, as advanced disease is less responsive to sight-preserving interventions like corneal cross-linking. Geographic factors compound these disparities, with patients in socially disadvantaged areas demonstrating reduced rates of accessing advanced treatments. The concentration of sophisticated diagnostic equipment in tertiary care centers creates additional barriers for populations lacking transportation, insurance coverage, or proximity to specialized services. These systemic inequities result in a two-tiered system where early detection, and optimal visual outcomes become privileges rather than universal healthcare standards.

### 4.5. Multimodal Approach and Clinical Integration

The evidence supports a multimodal diagnostic approach that combines the strengths of various imaging modalities while mitigating their individual limitations. Corneal topography provides accessible screening capabilities, tomography offers comprehensive structural analysis, optical coherence tomography reveals epithelial changes, and biomechanical assessment evaluates functional integrity. The integration of these modalities, particularly when enhanced by artificial intelligence algorithms, creates a diagnostic framework that maximizes sensitivity while maintaining specificity. This comprehensive approach is particularly valuable in managing high-risk populations, including patients with family history of keratoconus, those with allergic conditions, and individuals engaging in frequent eye rubbing. Early identification in these populations enables proactive monitoring and prompt intervention, preventing the visual deterioration that accompanies advanced disease.

### 4.6. Future Directions

The trajectory of keratoconus diagnostic technology suggests continued improvement in early detection capabilities, with corresponding benefits for visual outcomes. Emerging technologies such as Brillouin microscopy offer novel insights into corneal biomechanical properties, potentially identifying at-risk corneas before any morphological changes occur. Similarly, advances in artificial intelligence and machine learning promise to further enhance diagnostic accuracy and accessibility.

The development of portable, cost-effective diagnostic tools represents a critical need for expanding early detection capabilities to underserved populations. Such innovations could democratize access to advanced diagnostic capabilities, ensuring that the benefits of early detection and improved visual outcomes reach all patients regardless of socioeconomic status or geographic location.

### 4.7. Clinical Practice Implications

The evidence presented in this review establishes clear clinical imperatives for implementing comprehensive early detection strategies. The superior visual outcomes associated with early intervention justify the resource allocation required for advanced diagnostic equipment and specialized training. Healthcare systems must prioritize early detection programs, particularly in high-risk populations, to maximize the preservation of functional vision and minimize the long-term burden of advanced keratoconus. The integration of genetic screening and environmental risk factor assessment into routine clinical practice offers additional opportunities for early identification. Patients with positive family history or modifiable risk factors such as chronic eye rubbing represent ideal candidates for enhanced screening protocols, enabling intervention before significant visual compromise occurs.

In conclusion, the relationship between early keratoconus detection and visual outcomes represents one of the most compelling arguments for investing in advanced diagnostic capabilities and comprehensive screening programs. The evidence consistently demonstrates that earlier detection enables more effective intervention, resulting in better visual outcomes, reduced need for invasive procedures, and improved quality of life for affected patients. As diagnostic technologies continue to evolve and become more accessible, the potential for preserving vision through early detection will only continue to expand.

## 5. Conclusions

Early detection of keratoconus is essential for preventing irreversible vision loss and minimizing the need for advanced surgical interventions. A multimodal diagnostic approach, combining imaging and biomechanical assessment, improves accuracy in identifying subclinical disease. Ensuring equitable access to timely diagnosis and treatment is critical for optimizing patient outcomes. These insights support a proactive, personalized model of care that can preserve vision and enhance quality of life for individuals at risk of keratoconus.

## 6. Forward Trajectories

### 6.1. Development of Portable, Point-of-Care Technologies

Transforming early keratoconus detection requires miniaturized imaging and biomechanical devices that can be deployed in community clinics, school screenings, and remote settings. Handheld Scheimpflug or OCT platforms, coupled with simplified air-puff tonometry modules, would enable non-specialist practitioners to identify ectatic changes and refer patients for definitive evaluation.

### 6.2. Advanced Biomarker Discovery

Beyond structural and biomechanical metrics, molecular biomarkers in tear fluid, such as cytokine profiles or microRNA signatures, offer the promise of non-invasive early detection. Prospective studies should characterize proteomic and metabolomic patterns in at-risk corneas and evaluate their correlation with imaging findings and genetic predisposition.

### 6.3. Integration of Multimodal Data Through Machine Learning

While artificial intelligence has demonstrated high accuracy in retrospective analyses, the next step is to build prospective, longitudinal models that incorporate serial imaging, biomechanical measurements, genetic variants, environmental exposures, and clinical parameters. These predictive algorithms could generate individualized risk scores, guide surveillance intervals, and inform personalized intervention thresholds.

### 6.4. Genotype–Phenotype Correlation and Cost-Effective Genetic Screening

Comprehensive genome-wide association studies across diverse populations are needed to validate and expand the repertoire of keratoconus susceptibility loci. Coupled with health-economic analyses, this work could support the implementation of targeted genetic panels in families with a history of ectatic disease, enabling preemptive monitoring.

### 6.5. Tele-Ophthalmology Networks and Public-Health Programs

Embedding AI-enabled diagnostic software into smartphone-based slit-lamp adapters or fundus cameras can bring keratoconus screening into primary-care and school-based vision programs. Policymakers and healthcare providers should collaborate to subsidize these initiatives in underserved areas, ensuring that advances in early detection translate into real-world reductions in advanced disease burden.

### 6.6. Health-Systems Research on Access and Outcomes

Finally, implementation science studies must evaluate how best to integrate novel diagnostics and treatment pathways into varied clinical environments. Metrics should include time to diagnosis, cross-linking uptake, progression rates, patient—reported visual function, and cost-effectiveness. Such evidence will guide policymakers in allocating resources to maximize both clinical impact and equity.

By pursuing these avenues—technological innovation, biomarker validation, sophisticated analytics, and equitable delivery—future efforts will move keratoconus care from late-stage management to true prevention, safeguarding vision for patients worldwide.

## Data Availability

No new data were created or analyzed in this study.

## Abbreviations

The following abbreviations are used in this manuscript:

AS-OCT	anterior segment optical coherence tomography
KC	Keratoconus
CXL	Corneal Collagen Cross-linking

## References

[1] Mohammadpour M., Heidari Z., Hashemi H. (2017). Updates on managements for Keratoconus. J. Curr. Ophthalmol..

[2] Kreps E.O., Claerhout I., Koppen C. (2020). Diagnostic patterns in keratoconus. Contact Lens Anterior Eye.

[3] Asimellis G., Kaufman E.J. (2024). Keratoconus.

[4] Salman A., Darwish T., Ghabra M., Kailani O., Haddeh Y., Askar M., Ali A., Ali A., Alhassan S. (2022). Prevalence of keratoconus in a Population-Based study in Syria. J. Ophthalmol..

[5] Al-Amri A.M. (2018). Prevalence of keratoconus in a refractive surgery population. J. Ophthalmol..

[6] Gordon-Shaag A., Millodot M., Shneor E., Liu Y. (2015). The genetic and environmental factors for keratoconus. BioMed Res. Int..

[7] Mousa R.M., Saif M.Y.S., Said M.A.E., Taher R.M.M. (2024). Prevalence of keratoconus and characteristics of refractive errors in First-Degree relatives of patients with keratoconus among Egyptians. Cornea.

[8] Santodomingo-Rubido J., Carracedo G., Suzaki A., Villa-Collar C., Vincent S.J., Wolffsohn J.S. (2022). Keratoconus: An updated review. Contact Lens Anterior Eye.

[9] Vohra V., Tuteja S., Gurnani B., Chawla H. (2023). Collagen Cross Linking for Keratoconus.

[10] Pérez J.F., Marcos A.V., Peña F.J.M. (2014). Early diagnosis of keratoconus: What difference is it making?. Br. J. Ophthalmol..

[11] Althomali T.A., Al-Qurashi I.M., Al-Thagafi S.M., Mohammed A., Almalki M. (2017). Prevalence of keratoconus among patients seeking laser vision correction in Taif area of Saudi Arabia. Saudi J. Ophthalmol..

[12] Bui A.D., Truong A., Pasricha N., Indaram M. (2023). Keratoconus diagnosis and treatment: Recent advances and future directions. Clin. Ophthalmol..

[13] Olivo-Payne A., Abdala-Figuerola A., Hernandez-Bogantes E., Pedro-Aguilar L., Chan E., Godefrooij D. (2019). Optimal management of pediatric keratoconus: Challenges and solutions. Clin. Ophthalmol..

[14] Buzzonetti L., Bohringer D., Liskova P., Lang S., Valente P. (2020). Keratoconus in Children: A literature review. Cornea.

[15] Christensen M., Kartchner J., Giegengack M., Thompson A. (2024). A comparison of Black and Non-Black patients in the presentation and treatment of Keratoconus. Clin. Ophthalmol..

[16] Cavas-Martínez F., De La Cruz Sánchez E., Martínez J.N., Cañavate F.J.F., Fernández-Pacheco D.G. (2016). Corneal topography in keratoconus: State of the art. Eye Vis..

[17] Sridhar U., Tripathy K. (2023). Corneal Topography.

[18] Wegener A., Laser-Junga H. (2009). Photography of the anterior eye segment according to Scheimpflug’s principle: Options and limitations—A review. Clin. Exp. Ophthalmol..

[19] Motlagh M.N., Moshirfar M., Murri M.S., Skanchy D.F., Momeni-Moghaddam H., Ronquillo Y.C., Hoopes P.C. (2019). Pentacam^®^ Corneal Tomography for screening of refractive Surgery Candidates: A Review of the Literature, Part I. Med. Hypothesis Discov. Innov. Ophthalmol..

[20] Du X.L., Chen M., Xie L.X. (2015). Correlation of basic indicators with stages of keratoconus assessed by Pentacam tomography. Int. J. Ophthalmol..

[21] Ramos-López D., Martínez-Finkelshtein A., Castro-Luna G.M., Burguera-Gimenez N., Vega-Estrada A., Piñero D., Alió J.L. (2013). Screening subclinical keratoconus with Placido-Based Corneal indices. Optom. Vis. Sci..

[22] Ortiz-Toquero S., Rodriguez G., De Juan V., Martin R. (2014). Repeatability of Placido-Based corneal topography in keratoconus. Optom. Vis. Sci..

[23] Lee Y., Kim T.H., Paik H.J., Kim D.H. (2024). Artificial tear Instillation-Induced changes in corneal topography. Bioengineering.

[24] Gomes Ja P., Tan D., Rapuano C.J., Belin M.W., Ambrósio R., Guell J.L., Malecaze F., Nishida K., Sangwan V.S., Group of Panelists for the Global Delphi Panel of Keratoconus and Ectatic Diseases (2015). Global consensus on keratoconus and Ectatic diseases. Cornea.

[25] Toprak I., Cavas F., Velázquez J.S., Del Barrio J.L.A., Alio J.L. (2020). Subclinical keratoconus detection with three-dimensional (3-D) morphogeometric and volumetric analysis. Acta Ophthalmol..

[26] Bdour M.A., Sabbagh H.M., Jammal H.M. (2024). Multi-modal imaging for the detection of early keratoconus: A narrative review. Eye Vis..

[27] Thulasidas M., Teotia P. (2020). Evaluation of corneal topography and tomography in fellow eyes of unilateral keratoconus patients for early detection of subclinical keratoconus. Indian J. Ophthalmol..

[28] Balal S., Cai Y., Kandakji L., Liu S., Mullholand P.J., Leucci M., Pontikos N., Gore D., Allan B. (2025). Establishing the ground truth for keratoconus progression: Combining repeated measures and adapting precision limits to disease severity in tomography. J. Cataract. Refract. Surg..

[29] Shi Y. (2016). Strategies for improving the early diagnosis of keratoconus. Clin. Optom..

[30] Martin R. (2018). Cornea and anterior eye assessment with placido-disc keratoscopy, slit scanning evaluation topography and scheimpflug imaging tomography. Indian J. Ophthalmol..

[31] Golan O., Hwang E.S., Lang P., Santhiago M.R., Abulafia A., Touboul D., Krauthammer M., Smadja D. (2018). Differences in posterior corneal features between normal corneas and subclinical keratoconus. J. Refract. Surg..

[32] Maeno S., Koh S., Inoue R., Oie Y., Maeda N., Jhanji V., Nishida K. (2022). Fourier analysis on irregular corneal astigmatism using optical coherence tomography in various severity stages of keratoconus. Am. J. Ophthalmol..

[33] Han S.B., Liu Y.C., Noriega K.M., Mehta J.S. (2016). Applications of anterior segment optical coherence tomography in cornea and ocular surface diseases. J. Ophthalmol..

[34] Kanellopoulos A.J., Asimellis G. (2014). Anterior-Segment optical coherence tomography investigation of corneal deturgescence and epithelial remodeling after DSAEK. Cornea.

[35] Ramos J.L.B., Li Y., Huang D. (2008). Clinical and research applications of anterior segment optical coherence tomography—A review. Clin. Exp. Ophthalmol..

[36] Nowinska A.K., Teper S.J., Janiszewska D.A., Lyssek-Boron A., Dobrowolski D., Koprowski R., Wylegala E. (2015). Comparative Study of Anterior Eye Segment Measurements with Spectral Swept-Source and Time-Domain Optical Coherence Tomography in Eyes with Corneal Dystrophies. BioMed Res. Int..

[37] Asam J.S., Polzer M., Tafreshi A., Hirnschall N., Findl O. (2019). Anterior segment OCT. Springer eBooks.

[38] Serrano J.E., Jaimes C.P.T., Lucas C.E.M., Valero D.R., Martínez A.M., Toldos J.J.M. (2021). Intraobserver repeatability of tomographic, pachymetric, and anatomical measurements in healthy eyes using a new Swept-Source Optical Coherence topographer. Cornea.

[39] Feng Y., Reinstein D.Z., Nitter T., Archer T.J., McAlinden C., Chen X., Bertelsen G., Utheim T.P., Stojanovic A. (2022). Heidelberg Anterion Swept-Source OCT Corneal Epithelial Thickness Mapping: Repeatability and Agreement with Optovue Avanti. J. Refract. Surg..

[40] Schiano-Lomoriello D., Hoffer K.J., Abicca I., Savini G. (2021). Repeatability of automated measurements by a new anterior segment optical coherence tomographer and biometer and agreement with standard devices. Sci. Rep..

[41] Fişuş A.D., Hirnschall N.D., Ruiss M., Pilwachs C., Georgiev S., Findl O. (2021). Repeatability of 2 swept-source OCT biometers and 1 optical low-coherence reflectometry biometer. J. Cataract. Refract. Surg..

[42] Tañá-Rivero P., Aguilar-Córcoles S., Ruiz-Mesa R., Montés-Micó R. (2020). Repeatability of whole-cornea measurements using a new swept-source optical coherence tomographer. Eur. J. Ophthalmol..

[43] Kim K.Y., Choi G.S., Kang M.S., Kim U.S. (2020). Comparison study of the axial length measured using the new swept-source optical coherence tomography ANTERION and the partial coherence interferometry IOL Master. PLoS ONE.

[44] Chan P.P.M., Lai G., Chiu V., Chong A., Yu M., Leung C.K.S. (2020). Anterior chamber angle imaging with swept-source optical coherence tomography: Comparison between CASIAII and ANTERION. Sci. Rep..

[45] Niazi S., Jiménez-García M., Findl O., Gatzioufas Z., Doroodgar F., Shahriari M.H., Javadi M.A. (2023). Keratoconus diagnosis: From Fundamentals to Artificial intelligence: A Systematic Narrative review. Diagnostics.

[46] Itoi M., Kitazawa K., Yokota I., Wakimasu K., Cho Y., Nakamura Y., Hieda O., Teramukai S., Kinoshita S., Sotozono C. (2020). Anterior and posterior ratio of corneal surface areas: A novel index for detecting early stage keratoconus. PLoS ONE.

[47] Scuderi L., Anselmi G., Greco A., Abdolrahimzadeh B., Costa M.C., Scuderi G. (2021). Early identification of keratoconus using pachymetric indexes obtained with spectral domain optical coherence tomography. Clin. Ter..

[48] Tang M., Chen A., Li Y., Huang D. (2010). Corneal power measurement with Fourier-domain optical coherence tomography. J. Cataract. Refract. Surg..

[49] Naujokaitis T., Khoramnia R., Friedrich M., Son H.S., Auffarth G.U., Augustin V.A. (2024). Inter-zonal epithelial thickness differences for early keratoconus detection using optical coherence tomography. Eye.

[50] Li Y., Chamberlain W., Tan O., Brass R., Weiss J.L., Huang D. (2016). Subclinical keratoconus detection by pattern analysis of corneal and epithelial thickness maps with optical coherence tomography. J. Cataract. Refract. Surg..

[51] Kenia V.P., Kenia R.V., Maru S., Pirdankar O.H. (2023). Role of corneal epithelial mapping, Corvis biomechanical index, and artificial intelligence-based tomographic biomechanical index in diagnosing spectrum of keratoconus. Oman J. Ophthalmol..

[52] De Stefano V.S., Dupps W.J. (2017). Biomechanical diagnostics of the cornea. Int. Ophthalmol. Clin..

[53] Heidari Z., Hashemi H., Mohammadpour M., Amanzadeh K., Fotouhi A. (2021). Evaluation of corneal topographic, tomographic and biomechanical indices for detecting clinical and subclinical keratoconus: A comprehensive three-device study. Int. J. Ophthalmol..

[54] Terai N., Raiskup F., Haustein M., Pillunat L.E., Spoerl E. (2012). Identification of biomechanical properties of the cornea: The Ocular Response Analyzer. Curr. Eye Res..

[55] Ortiz D., Piñero D., Shabayek M.H., Arnalich-Montiel F., Alió J.L. (2007). Corneal biomechanical properties in normal, post-laser in situ keratomileusis, and keratoconic eyes. J. Cataract. Refract. Surg..

[56] Esporcatte L.P.G., Salomão M.Q., Lopes B.T., Vinciguerra P., Vinciguerra R., Roberts C., Elsheikh A., Dawson D.G., Ambrósio R. (2020). Biomechanical diagnostics of the cornea. Eye Vis..

[57] Fontes B.M., Ambrósio R., Jardim D., Velarde G.C., Nosé W. (2010). Corneal biomechanical metrics and anterior segment parameters in mild keratoconus. Ophthalmology.

[58] Bao F., Geraghty B., Wang Q., Elsheikh A. (2016). Consideration of corneal biomechanics in the diagnosis and management of keratoconus: Is it important?. Eye Vis..

[59] Ren S., Xu L., Fan Q., Gu Y., Yang K. (2021). Accuracy of new Corvis ST parameters for detecting subclinical and clinical keratoconus eyes in a Chinese population. Sci. Rep..

[60] Randleman J.B., Zhang H., Asroui L., Tarib I., Dupps W.J., Scarcelli G. (2023). Subclinical keratoconus detection and characterization using Motion-Tracking brillouin microscopy. Ophthalmology.

[61] Shao P., Eltony A.M., Seiler T.G., Tavakol B., Pineda R., Koller T., Seiler T., Yun S.-H. (2019). Spatially-resolved Brillouin spectroscopy reveals biomechanical abnormalities in mild to advanced keratoconus in vivo. Sci. Rep..

[62] Seiler T.G., Shao P., Eltony A., Seiler T., Yun S.H. (2019). Brillouin spectroscopy of normal and keratoconus corneas. Am. J. Ophthalmol..

[63] Huo Y., Chen X., Khan G.A., Wang Y. (2023). Corneal biomechanics in early diagnosis of keratoconus using artificial intelligence. Graefe S Arch. Clin. Exp. Ophthalmol..

[64] Vandevenne M.M., Favuzza E., Veta M., Lucenteforte E., Berendschot T., Mencucci R., Nuijts R.M.M.A., Virgili G., Dickman M.M. (2021). Artificial intelligence for detecting keratoconus. Cochrane Libr..

[65] Goodman D., Zhu A.Y. (2024). Utility of artificial intelligence in the diagnosis and management of keratoconus: A systematic review. Front. Ophthalmol..

[66] Mourgues E., Saunier V., Smadja D., Touboul D., Saunier V. (2024). Forme Fruste Keratoconus Detection with OCT Corneal Topography Using Artificial Intelligence Algorithms. J. Cataract. Refract. Surg..

[67] Hashemi H., Doroodgar F., Niazi S., Khabazkhoob M., Heidari Z. (2023). Comparison of different corneal imaging modalities using artificial intelligence for diagnosis of keratoconus: A systematic review and meta-analysis. Graefe S Arch. Clin. Exp. Ophthalmol..

[68] Nguyen T., Ong J., Masalkhi M., Waisberg E., Zaman N., Sarker P., Aman S., Lin H., Luo M., Ambrosio R. (2024). Artificial intelligence in corneal diseases: A narrative review. Contact Lens Anterior Eye.

[69] Shanthi S., Aruljyothi L., Balasundaram M.B., Janakiraman A., Nirmaladevi K., Pyingkodi M. (2021). Artificial intelligence applications in different imaging modalities for corneal topography. Surv. Ophthalmol..

[70] Afifah A., Syafira F., Afladhanti P.M., Dharmawidiarini D. (2024). Artificial intelligence as diagnostic modality for keratoconus: A systematic review and meta-analysis. J. Taibah Univ. Med. Sci..

[71] Kamiya K., Ayatsuka Y., Kato Y., Fujimura F., Takahashi M., Shoji N., Mori Y., Miyata K. (2019). Keratoconus detection using deep learning of colour-coded maps with anterior segment optical coherence tomography: A diagnostic accuracy study. BMJ Open.

[72] Tan Z., Chen X., Li K., Liu Y., Cao H., Li J., Jhanji V., Zou H., Liu F., Wang R. (2022). Artificial Intelligence–Based diagnostic model for detecting keratoconus using videos of corneal force deformation. Transl. Vis. Sci. Technol..

[73] Issarti I., Consejo A., Jiménez-García M., Hershko S., Koppen C., Rozema J.J. (2019). Computer aided diagnosis for suspect keratoconus detection. Comput. Biol. Med..

[74] Lavric A., Valentin P. (2019). KeratoDetect: Keratoconus detection algorithm using convolutional neural networks. Comput. Intell. Neurosci..

[75] Ueda D., Shimazaki A., Miki Y. (2018). Technical and clinical overview of deep learning in radiology. Jpn. J. Radiol..

[76] Chan S., Reddy V., Myers B., Thibodeaux Q., Brownstone N., Liao W. (2020). Machine learning in Dermatology: Current applications, opportunities, and Limitations. Dermatol. Ther..

[77] Souza M.B., Medeiros F.W., Souza D.B., Garcia R., Alves M.R. (2010). Evaluation of machine learning classifiers in keratoconus detection from orbscan II examinations. Clinics.

[78] Kovács I., Miháltz K., Kránitz K., Juhász É., Takács Á., Dienes L., Gergely R., Nagy Z.Z. (2016). Accuracy of machine learning classifiers using bilateral data from a Scheimpflug camera for identifying eyes with preclinical signs of keratoconus. J. Cataract. Refract. Surg..

[79] Xie Y., Zhao L., Yang X., Wu X., Yang Y., Huang X., Liu F., Xu J., Lin L., Lin H. (2020). Screening Candidates for Refractive Surgery with Corneal Tomographic-Based Deep Learning. JAMA Ophthalmol..

[80] Zéboulon P., Debellemanière G., Bouvet M., Gatinel D. (2020). Corneal Topography Raw Data Classification Using a Convolutional Neural Network. Am. J. Ophthalmol..

[81] Dos Santos V.A., Schmetterer L., Stegmann H., Pfister M., Messner A., Schmidinger G., Garhofer G., Werkmeister R.M. (2019). CorneaNet: Fast segmentation of cornea OCT scans of healthy and keratoconic eyes using deep learning. Biomed. Opt. Express.

[82] Quanchareonsap W., Kasetsuwan N., Reinprayoon U., Piyacomn Y., Wungcharoen T., Jermjutitham M. (2024). Deep Learning Algorithm for Keratoconus Detection from Tomographic Maps and Corneal Biomechanics: A Diagnostic Study. J. Curr. Ophthalmol..

[83] Gairola S., Joshi P., Balasubramaniam A., Murali K., Kwatra N., Jain M. (2022). Keratoconus Classifier for Smartphone-based Corneal Topographer. Annu. Int. Conf. IEEE Eng. Med. Biol. Soc..

[84] Nusair O. (2025). Clinical applications of artificial intelligence in corneal diseases. Vision.

[85] Herber R., Pillunat L.E., Raiskup F. (2021). Development of a classification system based on corneal biomechanical properties using artificial intelligence predicting keratoconus severity. Eye Vis..

[86] Syta A., Podkowiński A., Chorągiewicz T., Karpiński R., Gęca J., Wróbel-Dudzińska D., E Jonak K., Głuchowski D., Maciejewski M., Rejdak R. (2025). Machine learning-assisted early detection of keratoconus: A comparative analysis of corneal topography and biomechanical data. Sci. Rep..

[87] Aatila M., Lachgar M., Hamid H., Kartit A. (2021). Keratoconus severity classification using features selection and machine learning algorithms. Comput. Math. Methods Med..

[88] Gideon Abou Said A., Gispets J., Shneor E. (2025). Strategies for Early Keratoconus Diagnosis: A Narrative Review of Evaluating Affordable and Effective Detection Techniques. J. Clin. Med..

[89] Lavric A., Anchidin L., Popa V., Al-Timemy A.H., Alyasseri Z., Takahashi H., Yousefi S., Hazarbassanov R.M. (2021). Keratoconus Severity Detection From Elevation, Topography and Pachymetry Raw Data Using a Machine Learning Approach. IEEE Access.

[90] Cao K., Verspoor K., Sahebjada S., Baird P.N. (2022). Accuracy of Machine Learning Assisted Detection of Keratoconus: A Systematic Review and Meta-Analysis. J. Clin. Med..

[91] Loukovitis E., Sfakianakis K., Syrmakesi P., Tsotridou E., Orfanidou M., Bakaloudi D.R., Stoila M., Kozei A., Koronis S., Zachariadis Z. (2018). Genetic Aspects of Keratoconus: A Literature Review Exploring Potential Genetic Contributions and Possible Genetic Relationships with Comorbidities. Ophthalmol. Ther..

[92] Lucas S.E.M., Burdon K.P. (2020). Genetic and environmental risk factors for keratoconus. Annu. Rev. Vis. Sci..

[93] Rong S.S., Ue S.T.M.A., Yu X.T., Ma L., Chu W.K., Chan T.C.Y., Wang Y.M., Young A.L., Pang C.P., Jhanji V. (2017). Genetic associations for keratoconus: A systematic review and meta-analysis. Sci. Rep..

[94] Moran S., Gomez L., Zuber K., Gatinel D. (2020). A Case-Control Study of Keratoconus risk Factors. Cornea.

[95] Weed K.H., MacEwen C.J., Giles T., Low J., McGhee C.N.J. (2007). The Dundee University Scottish Keratoconus study: Demographics, corneal signs, associated diseases, and eye rubbing. Eye.

[96] Hashemi H., Heydarian S., Hooshmand E., Saatchi M., Yekta A., Aghamirsalim M., Valadkhan M., Mortazavi M., Hashemi A., Khabazkhoob M. (2019). The Prevalence and Risk Factors for Keratoconus: A Systematic Review and Meta-Analysis. Cornea.

[97] Ahmad T.R., Kong A.W., Turner M.L., Barnett J., Kaur G., O’Brien K.S., Pasricha N.D., Indaram M. (2022). Socioeconomic correlates of keratoconus severity and progression. Cornea.

[98] Merdler I., Hassidim A., Sorkin N., Shapira S., Gronovich Y., Korach Z. (2015). Keratoconus and allergic diseases among Israeli adolescents between 2005 and 2013. Cornea.

[99] Naderan M., Rajabi M.T., Zarrinbakhsh P., Bakhshi A. (2017). Effect of allergic diseases on keratoconus severity. Ocul. Immunol. Inflamm..

[100] Gupta A.S., Yu Y., Orlin S.E., VanderBeek B.L. (2023). Real world socioeconomic determinants of corneal crosslinking in a national cohort. J. Cataract. Refract. Surg..

